# Supplementary material for: Prevalence, trends, and factors associated with maternal autonomy regarding healthcare, finances, and mobility in Bangladesh: Analysis of Demographic and Health Surveys 1999–2018
Source: PLOS Glob Public Health. 2024 Feb 2;4(2):e0002816. doi: 10.1371/journal.pgph.0002816 (PMC10836669; doi:10.1371/journal.pgph.0002816)
Supplement: S1 Fig — (DOCX) [file pgph.0002816.s001.docx]

**S1 Fig: Steps of data collection of Bangladesh Demographic and Health Survey 1999-2000, 2004, 2007, 2011, 2014, & 2017-18**


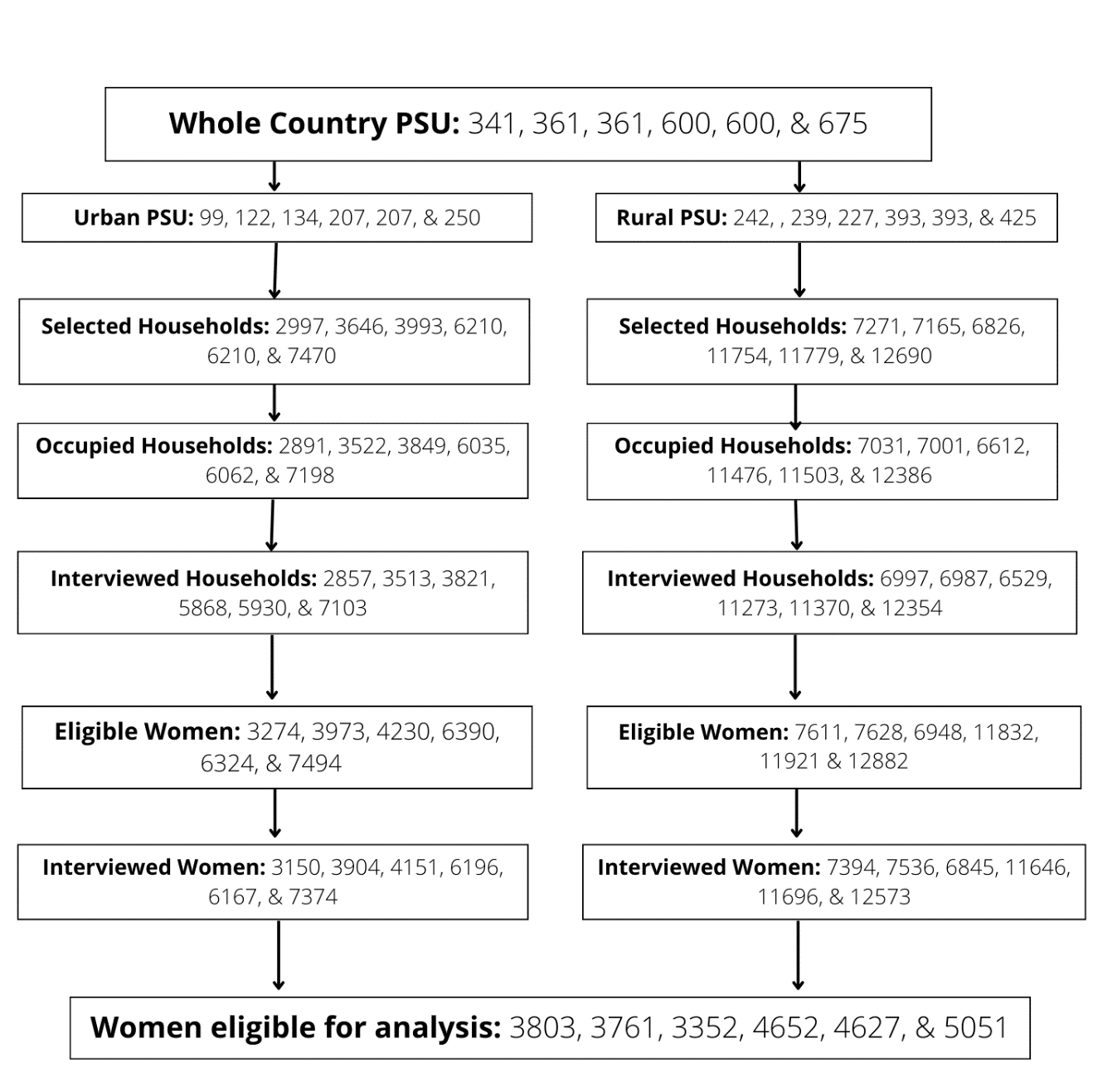


*Note: The numbers inside the box represent the units* in BDHS *1999-2000, 2004, 2007, 2011, 2014, & 2017-18, respectively. Women with at least one birth in past three years prior to the survey were eligible for analysis. PSU: Primary Sampling Uni*
