# Supplementary material for: Prevalence, trends, and factors associated with maternal autonomy regarding healthcare, finances, and mobility in Bangladesh: Analysis of Demographic and Health Surveys 1999–2018
Source: PLOS Glob Public Health. 2024 Feb 2;4(2):e0002816. doi: 10.1371/journal.pgph.0002816 (PMC10836669; doi:10.1371/journal.pgph.0002816)
Supplement: S1 Table — (DOCX) [file pgph.0002816.s002.docx]

**S1 Table: Study variables**

| **Variable Name** | **Definition and Categories** |
| --- | --- |
| Maternal age (in years): | Women reported their age. This ordinal variable was categorized as 15-19, 20-29, and 30-49 years. |
| Parity | It was an ordinal variable as well. Mothers reported the number of times they became pregnant in their life. It was categorized as primi (i.e., the first pregnancy), 2^nd^ to 3^rd^, and 4^th^ or more pregnancy. |
| Maternal education level | Women were asked about their education level. It was also an ordinal variable. This variable was categorized as no formal education, primary (i.e., up to 5 completed school years), secondary (i.e., up to 10 completed school years), and college or above. |
| Paternal education level | This is the education level of the father of a child. Was categorized as no formal education, primary, secondary, and college or above (see above). |
| Current work status | This was a binary variable. It was defined as whether the mother was involved in an occupation that results in earning money. |
| Religion | Women reported the religion they follow. As the vast majority (about 90%) of the people are Muslims, a binary variable was created as ‘Islam’ or ‘other’. |
| Household wealth status | DHS employs principal component analysis to calculate wealth status of household. First, basic household construction materials (i.e., materials used to construct the walls, roof, and floors), source of drinking water of the household members, availability of electricity, sanitation facilities, and other common household belongings were employed to construct wealth index score for each household. Then, it was stratified into quintiles to produce an ordinal variable: poorest, poorer, middle, richer, and richest. |
| Place of residence | This was a binary variable and described whether a study participant lived in a rural or urban region. Women living in a municipal or city corporation were considered as urban women; all other women were considered as rural women. |
| Division of residence | Division is the largest administrative unit of Bangladesh. Bangladesh had eight divisions during BDHS 2017-18 survey period: Barisal, Chittagong, Dhaka, Khulna, Mymensingh, Rajshahi, Rangpur, and Sylhet. Earlier surveys had a lower number of divisions. |
| Survey years | These are the periods when the BDHS were conducted: 1999-00, 2004, 2007, 2011, 2014, and 2017-18. Survey years were used for analysis of Aim 1 only. For other two aims, only BDHS 2017-18 data was used. |
| Survey cluster | This is the unique identification number of each cluster where the mothers lived during the survey. This variable was required for the multilevel data analysis. People in a cluster may have some common characteristics. |
| Survey weight | This was provided by BDHS for each survey administration. It was required to reflect the weighted prevalence estimates. The primary aim of BDHS is to provide nationally representative estimates. It also aims to show estimates for rural-urban regions and each of the administrative divisions separately. During the sample collection process, as some place or divisions may be oversampled (i.e., non-proportional allocation), weighting is required to correct those sampling errors. BDHS then reports the prevalence estimates for all indicators using these weights. BDHS uses two-stage stratified cluster design, therefore, the weights are obtained according to sampling probabilities for each stage and cluster separately. Then, household non-response are adjusted to construct household sample weight and individual non-response is adjusted to construct individual sample weight. |
| Primary sampling weight | This is the primary sampling unit of the survey. |
| Strata | This is required to set the sample in survey mode |
