# Supplementary material for: Prevalence, trends, and factors associated with maternal autonomy regarding healthcare, finances, and mobility in Bangladesh: Analysis of Demographic and Health Surveys 1999–2018
Source: PLOS Glob Public Health. 2024 Feb 2;4(2):e0002816. doi: 10.1371/journal.pgph.0002816 (PMC10836669; doi:10.1371/journal.pgph.0002816)
Supplement: S2 Table — (DOCX) [file pgph.0002816.s003.docx]

**S2 Table: Prevalence (95% confidence interval) and trends of high maternal autonomy by sociodemographic characteristics**

| **Variable** | **1999-00** | **2004** | **2007** | **2011** | **2014** | **2017-18** | **Mean change** |
| --- | --- | --- | --- | --- | --- | --- | --- |
| **Maternal age (in year)** | | | | | | | |
| 15-19 | 30.3 (27.0,33.8) | 26.3 (23.1,29.9) | 31.5 (27.7,35.6) | 32.6 (29.3,36.1) | 35.2 (30.6,40.1) | 35.8 (32.4,39.4) | **1.6** (0.5,2.6)** |
| 20-29 | 37.1 (34.9,39.3) | 31.3 (29.2,33.4) | 41.9 (39.5,44.4) | 39.7 (37.7,41.8) | 43.8 (41.4,46.3) | 54.3 (52.4,56.3) | **3.6*** (3.0,4.2)** |
| 30-49 | 41.1 (37.5,44.7) | 35.7 (32.1,39.4) | 48.5 (44.1,52.9) | 51.5 (47.7,55.3) | 55.4 (50.9,59.8) | 67.0 (63.7,70.0) | **5.6*** (4.6,6.5)** |
| **Maternal education level** | | | | | | | |
| No education | 35.3 (32.8,37.8) | 28.9 (26.2,31.7) | 41.0 (37.1,45.1) | 39.6 (35.7,43.5) | 46.0 (40.7,51.5) | 58.2 (52.1,64.2) | **3.7*** (2.6,4.9)** |
| Primary | 35.1 (32.1,38.2) | 31.4 (28.5,34.4) | 38.0 (34.6,41.5) | 37.6 (34.8,40.6) | 44.2 (40.4,48.0) | 56.9 (54.0,59.8) | **4.3*** (3.5,5.2)** |
| Secondary | 37.4 (33.9,40.9) | 31.4 (28.5,34.5) | 40.9 (37.8,44.0) | 40.1 (37.8,42.5) | 42.5 (39.6,45.4) | 50.1 (47.9,52.4) | **3.1*** (2.3,3.9)** |
| College/above | 53.8 (46.1,61.3) | 41.7 (35.0,48.8) | 54.6 (47.7,61.3) | 54.6 (48.9,60.2) | 50.9 (45.7,56.0) | 56.9 (53.2,60.6) | **1.6* (0.1,3.0)** |
| **Paternal education level** | | | | | | | |
| No education | 34.6 (32.1,37.2) | 29.9 (27.4,32.6) | 41.9 (38.5,45.3) | 40.2 (37.1,43.4) | 44.9 (40.5,49.3) | 63.0 (58.9,66.9) | **4.6*** (3.7,5.5)** |
| Primary | 34.6 (31.3,38.0) | 28.6 (25.7,31.8) | 35.2 (31.8,38.8) | 39.8 (36.9,42.8) | 43.1 (39.4,46.9) | 54.2 (51.6,56.9) | **4.5*** (3.7,5.3)** |
| Secondary | 36.7 (33.2,40.2) | 33.8 (30.6,37.2) | 41.7 (38.1,45.3) | 38.7 (35.9,41.6) | 43.4 (40.2,46.7) | 49.2 (46.5,51.8) | **2.7*** (1.9,3.5)** |
| College/above | 50.0 (44.9,55.2) | 35.9 (30.9,41.2) | 51.1 (45.5,56.7) | 46.2 (41.8,50.6) | 47.7 (42.8,52.6) | 58.0 (54.4,61.5) | **2.4*** (1.2,3.7)** |
| **Current work** | | | | | | | |
| No | 34.7 (32.9,36.4) | 30.3 (28.6,32.1) | 40.0 (37.8,42.1) | 39.5 (37.9,41.2) | 42.5 (40.3,44.8) | 50.2 (48.2,52.2) | **3.2*** (2.6,3.9)** |
| Yes | 45.6 (41.4,49.9) | 35.2 (31.1,39.5) | 44.3 (40.2,48.4) | 50.0 (44.3,55.7) | 50.0 (45.8,54.2) | 59.6 (57.1,61.9) | **3.8*** (2.9,4.8)** |
| **Wealth quintile** | | | | | | | |
| Poorest | 34.1 (30.8,37.4) | 30.5 (27.2,33.9) | 39.7 (35.5,44.1) | 41.1 (37.7,44.6) | 42.4 (37.9,47.0) | 57.3 (54.1,60.5) | **4.4*** (3.5,5.3)** |
| Poorer | 34.0 (30.5,37.6) | 26.9 (23.5,30.6) | 39.0 (34.9,43.2) | 37.8 (34.3,41.4) | 42.8 (38.5,47.1) | 52.5 (49.2,55.9) | **4.0*** (3.1,5.0)** |
| Middle | 37.5 (33.8,41.3) | 31.7 (28.1,35.5) | 36.3 (32.0,40.7) | 37.7 (34.3,41.3) | 44.9 (39.9,49.9) | 51.9 (48.3,55.5) | **3.3*** (2.3,4.4)** |
| Richer | 37.2 (33.2,41.4) | 30.8 (27.1,34.8) | 43.0 (38.6,47.4) | 39.1 (35.6,42.6) | 42.8 (38.6,47.2) | 53.3 (49.8,56.8) | **3.4*** (2.3,4.5)** |
| Richest | 41.6 (37.8,45.5) | 36.5 (32.9,40.3) | 47.4 (43.3,51.5) | 46.6 (42.8,50.4) | 48.9 (44.9,52.9) | 53.2 (49.6,56.6) | **2.7*** (1.6,3.9)** |
| **Place of residence** | | | | | | | |
| Urban | 38.8 (35.7,42.1) | 37.5 (34.4,40.7) | 45.9 (42.7,49.2) | 48.8 (45.6,52.0) | 48.5 (45.2,51.9) | 58.8 (56.0,61.5) | **4.0*** (3.0,5.0)** |
| Rural | 36.0 (34.1,37.8) | 29.5 (27.7,31.4) | 39.6 (37.3,41.9) | 37.9 (36.1,39.7) | 42.8 (40.4,45.3) | 51.8 (50.0,53.6) | **3.4*** (2.7,4.1)** |
| **Overall** | 36.4 (34.8,38.1) | 31.1 (29.5,32.7) | 41.0 (39.1,42.9) | 40.4 (38.8,42.0) | 44.3 (42.3,46.3) | 53.7 (52.1,55.2) | **3.7*** (3.1,4.2)** |

**: p<0.05, **: p<0.01, ***: p<0.001*
