# Supplementary material for: Prevalence, trends, and factors associated with maternal autonomy regarding healthcare, finances, and mobility in Bangladesh: Analysis of Demographic and Health Surveys 1999–2018
Source: PLOS Glob Public Health. 2024 Feb 2;4(2):e0002816. doi: 10.1371/journal.pgph.0002816 (PMC10836669; doi:10.1371/journal.pgph.0002816)
Supplement: S3 Table — (DOCX) [file pgph.0002816.s004.docx]

**S3 Table: Comparison of study sample by presence of any maternal autonomy, BDHS 1999-00 to 2017-18, % (n)^1^**

| **Variable** | | **Overall**  **(n = 25247)** | **Yes**  **(n = 18889)** | **No**  **(n = 6358)** | **p-values** |
| --- | --- | --- | --- | --- | --- |
| **Maternal age (in year)** | 15-19 | 20.5 (5179) | 18.2 (3447) | 27.2 (1732) | <0.001 |
|  | 20-29 | 59.5 (15034) | 60 (11338) | 58.1 (3696) |  |
|  | 30-49 | 19.9 (5034) | 21.7 (4104) | 14.6 (930) |  |
| **Number of children** | 1 | 34.9 (8816) | 32.7 (6169) | 41.6 (2647) | <0.001 |
|  | 2-3 | 45.8 (11565) | 48 (9061) | 39.4 (2504) |  |
|  | 4 or More | 19.3 (4866) | 19.4 (3658) | 19.0 (1208) |  |
| **Maternal education** | No education | 22.4 (5646) | 21 (3969) | 26.4 (1677) | <0.001 |
|  | Primary | 29.1 (7354) | 28.8 (5447) | 30 (1907) |  |
|  | Secondary | 39.5 (9967) | 40 (7559) | 37.9 (2407) |  |
|  | College/above | 9 (2280) | 10.1 (1912) | 5.8 (368) |  |
|  | No education | 29 (7272) | 27.8 (5236) | 32.5 (2037) | <0.001 |
| **Paternal education level** | Primary | 29.2 (7325) | 29.2 (5507) | 29 (1818) |  |
|  | Secondary | 28.7 (7215) | 28.7 (5410) | 28.8 (1805) |  |
|  | College/above | 13.1 (3288) | 14.2 (2676) | 9.8 (611) |  |
| **Current work status** | No | 78.8 (19880) | 76.9 (14518) | 84.3 (5361) | <0.001 |
|  | Yes | 21.2 (5364) | 23.1 (4368) | 15.7 (996) |  |
| **Religion** | Muslim | 91.4 (23069) | 91.3 (17252) | 91.5 (5818) | 0.75 |
|  | Other | 8.6 (2176) | 8.7 (1635) | 8.5 (540) |  |
| **Exposure to mass media** | Not exposed | 48.8 (12317) | 47.4 (8953) | 52.9 (3363) | <0.001 |
|  | Exposed | 51.2 (12931) | 52.6 (9935) | 47.1 (2995) |  |
| **Wealth quintile** | Poorest | 22.7 (5720) | 22 (4164) | 24.5 (1556) | <0.001 |
|  | Poorer | 20.4 (5157) | 20 (3779) | 21.7 (1379) |  |
|  | Middle | 19.6 (4943) | 19.3 (3647) | 20.4 (1296) |  |
|  | Richer | 19.1 (4827) | 19.4 (3656) | 18.4 (1171) |  |
|  | Richest | 18.2 (4601) | 19.3 (3643) | 15.1 (958) |  |
| **Place of residence** | Urban | 22.7 (5742) | 24.3 (4584) | 18.2 (1158) | <0.001 |
|  | Rural | 77.3 (19505) | 75.7 (14304) | 81.8 (5201) |  |
| **Division of residence** | Dhaka | 30.6 (7733) | 31 (5863) | 29.4 (1870) | <0.001 |
|  | Chittagong | 21.9 (5536) | 21.8 (4114) | 22.4 (1422) |  |
|  | Rajshahi | 16.5 (4178) | 16.8 (3174) | 15.8 (1004) |  |
|  | Khulna | 9.5 (2399) | 9.5 (1803) | 9.4 (597) |  |
|  | Barisal | 5.9 (1480) | 5.7 (1072) | 6.4 (409) |  |
|  | Sylhet | 8 (2015) | 7 (1325) | 10.9 (691) |  |
|  | Rangpur | 5.8 (1475) | 6.2 (1174) | 4.7 (301) |  |
|  | Mymensingh | 1.7 (431) | 1.9 (365) | 1 (66) |  |

*1.Weighted column percentages and weighted numbers, sample weights were provided with each BDHS*

*Abbreviation: BDHS: Bangladesh Demographic & Health Survey*
