# Supplementary material for: Prevalence, trends, and factors associated with maternal autonomy regarding healthcare, finances, and mobility in Bangladesh: Analysis of Demographic and Health Surveys 1999–2018
Source: PLOS Glob Public Health. 2024 Feb 2;4(2):e0002816. doi: 10.1371/journal.pgph.0002816 (PMC10836669; doi:10.1371/journal.pgph.0002816)
Supplement: S4 Table — (DOCX) [file pgph.0002816.s005.docx]

**S4 Table: Comparison of Study Sample by Level of Autonomy, BDHS 1999-00 to 2017-18, % (n)**

| **Variable** | **Overall**  **(N = 25247)** | **No**  **(N = 6358)** | **Low**  **(N = 8320)** | **High**  **(N = 10658)** | **p-value** |
| --- | --- | --- | --- | --- | --- |
| **Maternal age (in year)** | | | | | |
| 15-19 | 20.5 (5179) | 27.2 (1732) | 21.4 (1784) | 15.7 (1663) | <0.001 |
| 20-29 | 59.5 (15034) | 58.1 (3696) | 60 (4995) | 60 (6342) |  |
| 30-49 | 19.9 (5034) | 14.6 (930) | 18.5 (1541) | 24.2 (2563) |  |
| **Parity** | | | | | |
| 1 | 34.9 (8816) | 41.6 (2647) | 35.3 (2941) | 30.5 (3228) | <0.001 |
| 2-3 | 45.8 (11565) | 39.4 (2504) | 46 (3831) | 49.5 (5231) |  |
| 4 or More | 19.3 (4866) | 19 (1208) | 18.6 (1549) | 20 (2109) |  |
| **Maternal education level** | | | | | |
| No education | 22.4 (5646) | 26.4 (1677) | 22.1 (1838) | 20.2 (2131) | <0.001 |
| Primary | 29.1 (7354) | 30 (1907) | 29.1 (2422) | 28.6 (3025) |  |
| Secondary | 39.5 (9967) | 37.9 (2407) | 40.5 (3366) | 39.7 (4193) |  |
| College/above | 9 (2280) | 5.8 (368) | 8.3 (694) | 11.5 (1219) |  |
| **Paternal education level** | | | | | |
| No education | 29 (7272) | 32.5 (2037) | 28.1 (2330) | 27.6 (2905) | <0.001 |
| Primary | 29.2 (7325) | 29 (1818) | 30.2 (2507) | 28.5 (3001) |  |
| Secondary | 28.7 (7215) | 28.8 (1805) | 29 (2405) | 28.5 (3005) |  |
| College/above | 13.1 (3288) | 9.8 (611) | 12.6 (1045) | 15.5 (1631) |  |
| **Current work status** | | | | | |
| No | 78.8 (19880) | 84.3 (5361) | 79.9 (6652) | 74.4 (7866) | <0.001 |
| Yes | 21.2 (5364) | 15.7 (996) | 20.1 (1669) | 25.6 (2700) |  |
| **Religion** | | | | | |
| Muslim | 91.4 (23069) | 91.5 (5818) | 91.4 (7609) | 91.3 (9642) | 0.87 |
| Other | 8.6 (2176) | 8.5 (540) | 8.6 (712) | 8.7 (924) |  |
| **Mass media exposure** | | | | | |
| Not exposed | 48.8 (12317) | 52.9 (3363) | 47 (3913) | 47.7 (5040) | <0.001 |
| Exposed | 51.2 (12931) | 47.1 (2995) | 53 (4408) | 52.3 (5527) |  |
| **Wealth quintile** | | | | | |
| Poorest | 22.7 (5720) | 24.5 (1556) | 21.7 (1809) | 22.3 (2355) | <0.001 |
| Poorer | 20.4 (5157) | 21.7 (1379) | 20.9 (1740) | 19.3 (2039) |  |
| Middle | 19.6 (4943) | 20.4 (1296) | 19.7 (1639) | 19 (2008) |  |
| Richer | 19.1 (4827) | 18.4 (1171) | 19.6 (1629) | 19.2 (2027) |  |
| Richest | 18.2 (4601) | 15.1 (958) | 18.1 (1503) | 20.2 (2140) |  |
| **Place of residence** | | | | | |
| Urban | 22.7 (5742) | 18.2 (1158) | 21.9 (1819) | 26.2 (2766) | <0.001 |
| Rural | 77.3 (19505) | 81.8 (5201) | 78.1 (6502) | 73.8 (7802) |  |
| **Division of residence** | | | | | |
| Dhaka | 30.6 (7733) | 29.4 (1870) | 29.9 (2490) | 31.9 (3373) | <0.001 |
| Chittagong | 21.9 (5536) | 22.4 (1422) | 21.6 (1798) | 21.9 (2316) |  |
| Rajshahi | 16.5 (4178) | 15.8 (1004) | 18 (1499) | 15.9 (1676) |  |
| Khulna | 9.5 (2399) | 9.4 (597) | 9.9 (826) | 9.2 (977) |  |
| Barisal | 5.9 (1480) | 6.4 (409) | 6.1 (510) | 5.3 (562) |  |
| Sylhet | 8 (2015) | 10.9 (691) | 7.7 (640) | 6.5 (684) |  |
| Rangpur | 5.8 (1475) | 4.7 (301) | 5.6 (464) | 6.7 (710) |  |
| Mymensingh | 1.7 (431) | 1 (66) | 1.1 (95) | 2.6 (270) |  |

*Abbreviation: BDHS: Bangladesh Demographic & Health Survey*
