# Supplementary material for: Prevalence, trends, and factors associated with maternal autonomy regarding healthcare, finances, and mobility in Bangladesh: Analysis of Demographic and Health Surveys 1999–2018
Source: PLOS Glob Public Health. 2024 Feb 2;4(2):e0002816. doi: 10.1371/journal.pgph.0002816 (PMC10836669; doi:10.1371/journal.pgph.0002816)
Supplement: S5 Table — (DOCX) [file pgph.0002816.s006.docx]

**S5 Table: Factors associated with maternal autonomy, BDHS 1999-00 to 2017-18**

| **Variable** | **Unadjusted POR (95% CI)** | | **Adjusted POR (95% CI)^1^** | |
| --- | --- | --- | --- | --- |
|  | **Low vs**  **No (Ref)** | **High vs**  **No (Ref)** | **Low vs**  **No (Ref)** | **High vs**  **No (Ref)** |
| **Maternal age (year, Ref.: 15-19 years)** | | | | |
| 20-29 | 1.3*** (1.2,1.4) | 1.8*** (1.6,2.0) | 1.2** (1.1,1.3) | 1.4*** (1.3,1.6) |
| 30-49 | 1.6*** (1.5,1.9) | 3.0*** (2.6,3.4) | 1.7*** (1.5,2.0) | 2.5*** (2.2,2.9) |
| **Parity (Ref.: Primi)** | | | | |
| 2-3 | 1.4*** (1.3,1.5) | 1.4*** (1.3,1.5) | 1.3*** (1.2,1.5) | 1.5*** (1.4,1.7) |
| 4 or More | 1.2** (1.1,1.3) | 1.2** (1.1,1.3) | 1.1 (1.0,1.3) | 1.3** (1.1,1.5) |
| **Maternal education level (Ref.: No formal education)** | | | | |
| Primary | 1.1** (1.0,1.3) | 1.2*** (1.1,1.4) | 1.2** (1.1,1.3) | 1.3*** (1.2,1.4) |
| Secondary | 1.3*** (1.1,1.4) | 1.4*** (1.2,1.5) | 1.3*** (1.2,1.5) | 1.4*** (1.3,1.6) |
| College/above | 1.7*** (1.4,2.0) | 2.6*** (2.3,3.1) | 1.6*** (1.3,2.0) | 2.1*** (1.7,2.6) |
| **Paternal education level (Ref.: No formal education)** | | | | |
| Primary | 1.2*** (1.1,1.3) | 1.2** (1.0,1.3) | 1.1 (1.0,1.2) | 1.0 (0.9,1.1) |
| Secondary | 1.1** (1.0,1.3) | 1.2** (1.1,1.3) | 1.0 (0.9,1.1) | 1.0 (0.9,1.1) |
| College/above | 1.5*** (1.3,1.7) | 1.9*** (1.6,2.2) | 1.1 (0.9,1.3) | 1.1 (0.9,1.3) |
| **Current work status (Ref.: No)** | | | | |
| Yes | 1.4*** (1.2,1.5) | 1.9*** (1.7,2.1) | 1.2*** (1.1,1.4) | 1.5*** (1.3,1.6) |
| **Religion (Ref.: Muslim)** | | | | |
| Other | 1.0 (0.9,1.1) | 1.0 (0.9,1.2) |  |  |
| **Mass media exposure (Ref: Not exposed)** | | | | |
| Exposed | 1.3*** (1.2,1.4) | 1.2*** (1.1,1.3) | 1.2*** (1.1,1.3) | 1.1* (1.0,1.2) |
| **Wealth quintile (Ref.: Poorest)** | | | | |
| Poorer | 1.1 (0.9,1.2) | 1.0 (0.9,1.1) | 1.0 (0.9,1.1) | 0.9 (0.8,1.0) |
| Middle | 1.1 (1.0,1.2) | 1.0 (0.9,1.1) | 1.0 (0.8,1.1) | 0.9 (0.8,1.1) |
| Richer | 1.2* (1.0,1.3) | 1.1* (1.0,1.3) | 1.0 (0.8,1.1) | 0.9 (0.8,1.1) |
| Richest | 1.3*** (1.2,1.5) | 1.5*** (1.3,1.7) | 1.0 (0.8,1.1) | 0.9 (0.8,1.1) |
| **Place of residence (Ref.: Urban)** | | | | |
| Rural | 0.8*** (0.7,0.9) | 0.6*** (0.5,0.7) | 0.8** (0.8,0.9) | 0.7*** (0.6,0.8) |
| **Division of residence (Ref.: Dhaka)** | | | | |
| Chittagong | 0.9 (0.8,1.0) | 1.0 (0.8,1.2) | 0.9 (0.8,1.0) | 0.9 (0.8,1.1) |
| Rajshahi | 1.1 (1.0,1.3) | 1.0 (0.8,1.1) | 1.1 (1.0,1.3) | 1.1 (0.9,1.2) |
| Khulna | 1.0 (0.8,1.2) | 0.9 (0.7,1.1) | 1.0 (0.9,1.2) | 0.9 (0.8,1.1) |
| Barisal | 0.9 (0.8,1.1) | 0.8* (0.7,1.0) | 0.9 (0.8,1.1) | 0.8** (0.6,0.9) |
| Sylhet | 0.7*** (0.6,0.8) | 0.6*** (0.5,0.7) | 0.7*** (0.6,0.8) | 0.6*** (0.5,0.7) |
| Rangpur | 1.1 (0.9,1.4) | 1.3** (1.1,1.7) | 1.2 (1.0,1.5) | 1.2 (1.0,1.5) |
| Mymensingh | 1.1 (0.8,1.6) | 2.8*** (2.0,4.0) | 0.8 (0.5,1.2) | 1.5* (1.0,2.1) |
| **Survey period (Ref: 1999-00)** | | | | |
| 2004 | 0.9 (0.8,1.0) | 0.7*** (0.6,0.9) | 0.8* (0.7,1.0) | 0.7*** (0.6,0.8) |
| 2007 | 1.4*** (1.2,1.6) | 1.4*** (1.2,1.7) | 1.3** (1.1,1.5) | 1.3** (1.1,1.6) |
| 2011 | 0.9* (0.7,1.0) | 1.1 (0.9,1.3) | 0.8*** (0.7,0.9) | 1.0 (0.8,1.2) |
| 2014 | 0.8* (0.7,1.0) | 1.3** (1.1,1.5) | 0.7*** (0.6,0.9) | 1.0 (0.9,1.2) |
| 2017-18 | 1.5*** (1.3,1.7) | 2.7*** (2.3,3.1) | 1.3** (1.1,1.6) | 2.0*** (1.7,2.4) |

*Abbreviations: BDHS: Bangladesh Demographic & Health Survey; POR: Prevalence odds ratio; CI: confidence interval*

**: p<0.05, **: p<0.01, ***: p<0.001*

*1. Adjusted for all variables in the column*
