# Supplementary material for: Prevalence, trends, and factors associated with maternal autonomy regarding healthcare, finances, and mobility in Bangladesh: Analysis of Demographic and Health Surveys 1999–2018
Source: PLOS Glob Public Health. 2024 Feb 2;4(2):e0002816. doi: 10.1371/journal.pgph.0002816 (PMC10836669; doi:10.1371/journal.pgph.0002816)
Supplement: Attachment — Submitted filename: PGPH-D-23-01928.pdf [file pgph.0002816.s008.pdf]

PLOS Global Public Health

Prevalence, Trends, and Factors Associated with Maternal Autonomy Regarding Healthcare, Finances, and Mobility in Bangladesh: Analysis of Demographic and Health Surveys 1999-2018

--Manuscript Draft--

**Manuscript Number:**	PGPH-D-23-01928
**Article Type:**	Research Article
**Full Title:**	Prevalence, Trends, and Factors Associated with Maternal Autonomy Regarding Healthcare, Finances, and Mobility in Bangladesh: Analysis of Demographic and Health Surveys 1999-2018
**Short Title:**	Maternal autonomy in Bangladesh
**Corresponding Author:**	Gulam Muhammed Al Kibria<br>Johns Hopkins University Bloomberg School of Public Health<br>Baltimore, UNITED STATES
**Order of Authors:**	Gulam Muhammed Al Kibria
	Jennifer Albrecht, PhD
	Wendy Lane, MPH, MD
	Kristen Stafford, MPH, PhD
	Laundette Jones, PhD, MPH
	Roumen Vesselinov, MA, PhD
	Jon Mark Hirshon, MD, MPH, PhD
**Keywords:**	maternal autonomy; maternal decision-making; healthcare
**Abstract:**	Maternal autonomy is associated with improved healthcare utilization and outcomes for mothers and babies in low- and middle-income countries. We investigated the trends in the prevalence and factors associated with maternal autonomy in Bangladesh. This cross-sectional study analyzed the Bangladesh Demographic and Health Survey for years 1999-00, 2004, 2007, 2011, 2014, and 2017-18. Maternal autonomy was defined as the presence of at least one decision-making ability regarding healthcare, large household purchases, and freedom of mobility among 15-49-year-old mothers with at least one live birth in the past three years. We compared the samples based on the presence of at least one characteristic of autonomy and reported the trends in prevalence (95% confidence intervals (CIs)) across the survey years. Lastly, we performed multilevel logistic regression to report prevalence odds ratios (PORs) with 95% CIs for the factors associated with maternal autonomy. Variables investigated as potential factors included: maternal age, number of children, maternal education, paternal education, current work, religion, mass media exposure, wealth quintile, place and division of residence, and survey years. The prevalence of 'any' maternal autonomy was 72.0% (95% CI: 70.5-73.5) in 1999-00 and increased to 83.8% (95% CI: 82.7-84.9) in 2017-18. In adjusted analysis, mothers with older age, higher education, work outside the home, and mass media exposure had higher odds of autonomy than their counterparts (POR > 1, $p < 0.05$). For instance, compared to mothers without any formal education, the odds of autonomy were significantly ($p < 0.001$) higher among mothers with primary (adjusted POR: 1.2, 95% CI: 1.1-1.4), secondary (adjusted POR: 1.4, 95% CI: 1.2-1.6), and college/above (adjusted POR: 1.9, 95% CI: 1.6-2.2) education. While the level of maternal autonomy has increased over the past two decades, a substantial proportion of mothers still do not have autonomy. Expanding educational and earning opportunities may increase maternal autonomy. Further research is required to identify other factors and to understand the perspectives of husbands, in-laws, and other family members regarding the role of maternal autonomy.
**Additional Information:**	
**Question**	**Response**

Powered by Editorial Manager® and ProduXion Manager® from Aries Systems Corporation

**Financial Disclosure**<br><br>Enter a financial disclosure statement that describes the sources of funding for the work included in this submission and the role the funder(s) played. This includes grants and any commercial funding of the work or authors.<br><br>This statement will be typeset if the manuscript is accepted for publication.<br><br>*Please review the submission guidelines and the instructions link below for detailed requirements and guidance.*	The authors did not receive any funding to write this article
**Competing Interests**<br><br>On behalf of all authors, disclose any competing interests that could be perceived to bias this work.<br><br>This statement will be typeset if the manuscript is accepted for publication.<br><br>*Please review the instructions link below and PLOS Global Public Health's competing interests policy to determine what information must be disclosed at submission.*	None declared
**Data Availability**<br><br>Before publication, Authors are required to make fully available and without restriction all data underlying their findings. Please see our PLOS Data Policy page for detailed information on this policy.<br><br>A **Data Availability Statement**, detailing where the data can be accessed, is required at first submission. Insert your	Data is available to download upon approval from: https://dhsprogram.com/data/available-datasets.cfm

Data Availability Statement in the box below.<br><br>Please see the data reporting section of our submission guidelines for instructions on what you need to include in your Data Availability Statement.<br><br>This statement will be typeset if the manuscript is accepted for publication.<br><br>*PLOS allows rare exemptions to address legal and ethical concerns. If you have legal or ethical restrictions, please use the box below to detail these in full sentences for the Journal team to consider.*	

Aside from various edits related to further explanation of points raised by the authors, the need for more narrative explanation of results and background research, as well as additional references to the specific context [in the conclusion], the draft requires slight adjustment to the conclusions presented. Following on from the stated hypothesis, ("the prevalence of maternal autonomy has increased and that women with higher socioeconomic status (e.g., education or wealth) have higher autonomy than their counterparts") the data displayed in Table 3, particularly relating to "wealth quintile" demonstrate that none of the adjusted figures are significantly associated with maternal autonomy. However, in your conclusion section (page 12), you state that your findings confirm your hypothesis. While I can take this to understand that the first part of the hypothesis related to a prevalence increase has been supported, the second part of the hypothesis has clearly been demonstrated to have mixed results. It is critical that this is adequately addressed.

Additionally, the inclusion of the data on "lone maternal autonomy" is not thoroughly addressed anywhere other than the brief section in the results. Please consider adding more context to these findings as they represent an important facet of your argument.

Powered by Editorial Manager® and ProduXion Manager® from Aries Systems Corporation

Manuscript

Click here to access/download;Manuscript;Prev_Trends_Factors_Mat_Autono

Prevalence, Trends, and Factors Associated with Maternal Autonomy Regarding Healthcare, Finances, and Mobility in Bangladesh: Analysis of Demographic and Health Surveys 1999-2018

Gulam Muhammed Al Kibria[1*], MBBS, MSPH, PhD; Jennifer Albrecht[2], PhD; Wendy Lane[2], MD, MPH; Kristen A. Stafford[2], MPH, PhD; Laundette Jones[2], PhD, MPH; Roumen Vesselinov[2], MA, PhD; Jon Mark Hirshon[2,3,4], MD, MPH, PhD

Institutional Affiliations

1. Department of International Health, Johns Hopkins Bloomberg School of Public Health, Baltimore, MD 21205
2. Department of Epidemiology and Public Health, University of Maryland School of Medicine, Baltimore, MD 21201
3. Department of Emergency Medicine, University of Maryland School of Medicine, Baltimore, MD, 21201
4. Baltimore VA Medical Center, Baltimore, MD 21201.

Email: gkibria1@jhu.edu

Abstract

Maternal autonomy is associated with improved healthcare utilization and outcomes for mothers and babies in low- and middle-income countries. We investigated the trends in the prevalence and factors associated with maternal autonomy in Bangladesh. This cross-sectional study analyzed the Bangladesh Demographic and Health Survey for years 1999-00, 2004, 2007, 2011, 2014, and 2017-18. Maternal autonomy was defined as the presence of at least one decision-making ability regarding healthcare, large household purchases, and freedom of mobility among 15-49-year-old mothers with at least one live birth in the past three years. We compared the samples based on the presence of at least one characteristic of autonomy and reported the trends in prevalence (95% confidence intervals (CIs)) across the survey years. Lastly, we performed multilevel logistic regression to report prevalence odds ratios (PORs) with 95% CIs for the factors associated with maternal autonomy. Variables investigated as potential factors included: maternal age, number of children, maternal education, paternal education, current work, religion, mass media exposure, wealth quintile, place and division of residence, and survey years. The prevalence of 'any' maternal autonomy was 72.0% (95% CI: 70.5-73.5) in 1999-00 and increased to 83.8% (95% CI: 82.7-84.9) in 2017-18. In adjusted analysis, mothers with older age, higher education, work outside the home, and mass media exposure had higher odds of autonomy than their counterparts (POR > 1, $p < 0.05$). For instance, compared to mothers without any formal education, the odds of autonomy were significantly ($p < 0.001$) higher among mothers with primary (adjusted POR: 1.2, 95% CI: 1.1-1.4), secondary (adjusted POR: 1.4, 95% CI: 1.2-1.6), and college/above (adjusted POR: 1.9, 95% CI: 1.6-2.2) education. While the level of maternal autonomy has increased over the past two decades, a substantial proportion of mothers still do not have autonomy. Expanding educational and earning opportunities may increase maternal autonomy. Further research is required to identify other factors and to understand the perspectives of husbands, in-laws, and other family members regarding the role of maternal autonomy.

Keywords: Maternal autonomy; maternal decision-making; healthcare

Main Text

Introduction

Maternal autonomy is an indicator of overall socioeconomic development. It is often measured as a combination of several dimensions of decision-making ability regarding healthcare, finances, and mobility. It reflects the ability and self-confidence of women to overcome external barriers through their interaction with husbands, family members, and other stakeholders [1–3]. Studies from Bangladesh and other low- and middle-income countries (LMICs) have reported that maternal autonomy is associated with improved healthcare utilization and outcomes of mothers and children. Haider et al. (2017) analyzed Bangladesh Demographic and Health Survey (BDHS) 2011 data and found a nearly 50% higher prevalence of at least four antenatal care visits, institutional delivery, and neonatal postnatal care among women with autonomy in Bangladesh [4]. Another study by Haque et al. (2012) had similar results [5]. Sharma and Kader (2013) found 40% higher odds of low birth weight among children of mothers with lower autonomy compared to those with higher autonomy [6]. Overall, these studies demonstrated that higher maternal autonomy could increase healthcare utilization and decrease maternal/neonatal complications.

In recent decades, significant socioeconomic developments have taken place in Bangladesh, including increases in education levels, employment, and per-capita income [7,12,13]. Studies from other countries suggest that socioeconomic status (e.g., education or income) could impact maternal autonomy [14–17]. However, factors associated with maternal autonomy have not been studied in Bangladesh. Considering the significance of maternal autonomy in improving healthcare utilization and outcomes of mothers and children, examining factors associated with maternal autonomy across survey years could help identify women with low autonomy who might benefit from interventions to improve autonomy.

To capture data on women's empowerment in Bangladesh, BDHS reports the level of women's autonomy during each survey cycle [7]. However, the use of different definitions across survey periods yields different estimates of maternal autonomy. For instance, in BDHS 2017-18, women's autonomy was reported as the decision-making ability of women regarding large household purchases, healthcare, and

freedom of mobility. According to that report, approximately 60% of women aged 15-49 reported making these decisions alone or jointly with their husbands [7]. In BDHS 2011 and 2014, an additional variable (i.e., decision regarding child healthcare) was included, and it was found that 43% and 42% of women had autonomy, respectively [8,9]. The number of variables used to define autonomy was higher in 2004 and 1999-00 [10,11]. To track trends in the prevalence of maternal autonomy, it is important to use a common definition across all survey years.

The objectives of the present study are to investigate the trends in prevalence and factors associated with maternal autonomy using a common definition from a series of nationally representative surveys in Bangladesh. Based on previous reports, we hypothesize that the prevalence of maternal autonomy has increased and that women with higher socioeconomic status (e.g., education or wealth) have higher autonomy than their counterparts.

Methods

Data Source

In this cross-sectional study, we analyzed data from the BDHS for years 1999-00, 2004, 2007, 2011, 2014, and 2017-18. BDHS is a nationally representative household survey conducted every 3-4 years in Bangladesh. It used a two-stage stratified sampling method. Enumeration areas (EAs), also known as primary sampling units (PSUs), were selected in the first stage based on urban-rural distribution (i.e., probability proportional to size), followed by a systematic sample of households per EA in the next stage (S1 Fig). Sample sizes, target households, and completed interviews varied across survey years. Response rates ranged from 97-99%. This study used data from the women's questionnaire administered to women of reproductive age (i.e., 15-49-year-olds) who were ever married. We included women who had given birth within three years preceding the survey [6,14–17,24].

Ethics Statement:

The institutional review board (IRB) of the University of Maryland, Baltimore determined that this study meets the definition of non-human subject research and exempted it from IRB oversight (Approval Number: HP-00105503). Participants provided verbal informed consent to participate in the survey, if a participant was below 18 years of age, an adult household member provided consent.

Study Variables

Maternal Autonomy Women's autonomy was operationalized using three questions common over all survey years:

1) Person who usually decides on respondent's healthcare
2) Person who usually decides on major household purchases
3) Person who usually decides on visits to family and relatives

Five answer options were provided: respondent alone; respondent and husband/partner; husband/partner alone; someone else; and others [7]. Responses of making each decision 'alone' or 'jointly with husband/partner' were coded as 'have autonomy', all other options were coded as 'does not have autonomy'. By combining the three decision-making questions, a dichotomous variable representing autonomy was created: no (i.e., 'no' to all 3 questions, referent) and yes (i.e., 'yes' to at least 1 question). We also categorized autonomy by number of decision-making abilities: no (i.e., 'no' to all 3 questions), low (i.e., yes to 1-2 questions), and high (i.e., yes to all 3 questions) [7,18]. Finally, we also investigated whether mothers had lone autonomy (i.e., can take decisions without discussing with husbands).

Other Variables To identify factors associated with maternal autonomy, the following variables were examined: maternal age (i.e., 15-19, 20-29, and 30-49 years), number of children (i.e., '1', '2-3', and '4 or more'), maternal education (i.e., no formal education, primary, secondary, and college/above), paternal education (i.e., see maternal education), current work status (i.e., yes or no), wealth quintile (i.e., stratified as poorest, poorer, middle, richer, and richest), religion (i.e., Muslim or other), mass media exposure (i.e., yes or no), rural-urban place and division of residence, and survey years [4,7,19–25]. All the variables were based on self-report except the wealth quintile, which was based on principal component analysis of basic

household construction materials and other household belongings. S1 Table describes all the study variables.

Statistical analysis First, the sociodemographic and socioeconomic characteristics of the study participants were described by survey years. Variables were reported with weighted numbers of samples (n) and percentages (%). Differences in distributions across survey years were tested by Rao-Scott's chi-square tests. For each survey year, the weighted prevalence (and 95% confidence interval (CI)) of maternal autonomy was estimated for each factor. The trends in prevalence of maternal autonomy over survey years by selected characteristics were tested using Cochran-Armitage trend tests.

Next, the sample was compared by the presence of autonomy (any vs. none) using a similar approach. Lastly, unadjusted and adjusted multilevel logistic regression analyses were used to identify factors associated with maternal autonomy. Variables with p-values <0.05 in unadjusted analyses were added to the multivariable model. Multicollinearity among study variables was examined with variance inflation factor using a fake multiple linear regression model. Both unadjusted and adjusted prevalence odds ratios (POR) were reported with 95% CIs. As a secondary analysis, the prevalence, trends, and associated factors of at least two decision-making abilities were examined. The prevalence and trends of 'lone' maternal autonomy were also reported.

Data were analyzed using Stata 15.0 (statistical software, Stata Corporation, College Station, TX, USA). The 'svy' prefix in Stata command accommodates weighted survey data and accounts for survey design, coverage, and non-response when calculating estimates. [26].

Results

Sample Characteristics

Table 1: Maternal sociodemographic and socioeconomic characteristics by survey year, BDHS 1999-00 to 2017-18, % (n)[1]

Variable	1999-00 (n = 3803)	2004 (n=3761)	2007 (n=3352)	2011 (n=4652)	2014 (n=4627)	2017-18 (n=5051)	p-value

**Maternal age (in years)**							
15-19	22.2	22.3	21.1	19.6	21.0	17.9	**<0.001**
20-29	56.2	56.6	60.1	62.9	59.3	61.1	
30-49	21.7	21.1	18.8	17.5	19.7	21.0	
**Number of children**							
1	28.6	29.5	34.5	36.1	39.9	38.2	**<0.001**
2-3	43.7	42.9	43.9	46.7	46.3	49.6	
4 or More	27.7	27.5	21.6	17.1	13.8	12.2	
**Maternal education level**							
No education	45.3	35.5	23.8	17.6	14.2	6.3	**<0.001**
Primary	28.8	30.5	30.6	30.0	27.9	27.6	
Secondary	21.8	28.4	38.8	44.8	47.7	49.0	
College/above	4.1	5.6	6.8	7.5	10.2	17.1	
**Paternal education level**							
No education	43.4	39.2	33.0	27.6	23.8	13.7	**<0.001**
Primary	24.9	26.6	28.1	29.8	30.0	33.7	
Secondary	21.8	24.4	27.8	29.8	31.8	34.1	
College/above	9.8	9.8	11.0	12.8	14.4	18.5	
**Current work status**							
No	83.7	83.7	76.7	92.0	76.3	62.7	**<0.001**
Yes	16.3	16.3	23.3	8.0	23.7	37.3	
**Exposure to mass media**							
Not exposed	61.1	42.6	45.1	50.5	49.0	44.8	**<0.001**
Exposed	38.9	57.4	54.9	49.5	51.0	55.2	
**Religion**							
Muslim	89.3	92.5	91.6	91.2	91.7	91.9	0.52
Other	10.7	7.5	8.4	8.8	8.3	8.1	
**Wealth quintile**							
Poorest	25.6	24.8	21.1	22.8	21.7	20.6	**0.046**
Poorer	22.2	20.2	21.5	19.8	18.9	20.5	
Middle	20.0	20.5	19.2	19.7	19.1	19.2	
Richer	16.4	17.5	19.6	19.6	20.6	20.2	
Richest	15.8	16.9	18.6	18.1	19.7	19.5	
**Place of residence**							
Urban	16.6	19.9	21.6	23.0	26.1	26.8	**0.005**
Rural	83.4	80.1	78.4	77.0	73.9	73.2	
**Division of residence**							
Dhaka	30.5	30.9	31.8	30.5	35.3	25.6	**0.001**
Chittagong	21.8	21.6	21.8	23.3	21.8	21.2	
Rajshahi	24	22.3	22.6	13.3	10.0	11.6	
Khulna	10.3	11.1	9.4	9.5	8.0	9.2	
Barisal	6.2	6.0	6.1	5.6	5.8	5.7	
Sylhet	7.3	8.2	8.3	7.4	9.3	7.6	
Rangpur	NA	NA	NA	10.5	9.7	10.6	
Mymensingh	NA	NA	NA	NA	NA	8.5	

1.Weighted column percentages and weighted numbers (Column headers), sample weights were provided with each BDHS survey

Abbreviations: BDHS: Bangladesh Demographic & Health Survey; NA: Not applicable

Table 1 shows the sample characteristics by survey year. The weighted number of included mothers from BDHS 1999-00, 2004, 2007, 2011, 2014, and 2017-18 was 3803, 3761, 3352, 4652, 4627, and 5051, respectively. Most mothers (about 80%) were younger than 30 years old. Between 1999-00 and 2017-18, the proportion of mothers with at least four or more children declined from 27.7% (n = 1053) to 12.2% (n = 617). The proportion of mothers with education at or above college increased from 4.1% (n = 155) in 1999-00 to 17.1% (n = 864) in 2017-18. Similar increases in employment, paternal education, exposure to mass media, and urban residence were observed.

Prevalence and Trends of Maternal Autonomy

Figure 1: Prevalence (95% CI) of maternal autonomy by survey, BDHS 1999-00 to 2017-18

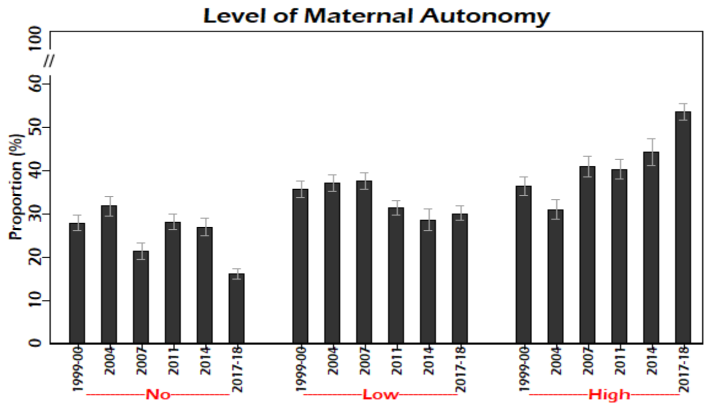

Abbreviation: BDHS: Bangladesh Demographic & Health Survey, CI: Confidence interval

The prevalence of maternal autonomy by survey year is shown in Figure 1. From 1999-00 to 2017-18, the overall prevalence of 'no' autonomy reduced from 28.0% (95% CI: 26.5-29.5) to 16.2% (95% CI: 15.1-17.3). The prevalence of 'high' (i.e., all 3 decisions) autonomy increased from 36.4% (95% CI: 34.8-38.1) in 1999-00 to 53.7% (95% CI: 52.1-55.2) in 2017-18.

Figure 2: Prevalence (95% CI) of each decision-making ability by survey, BDHS 1999-00 to 2017-18

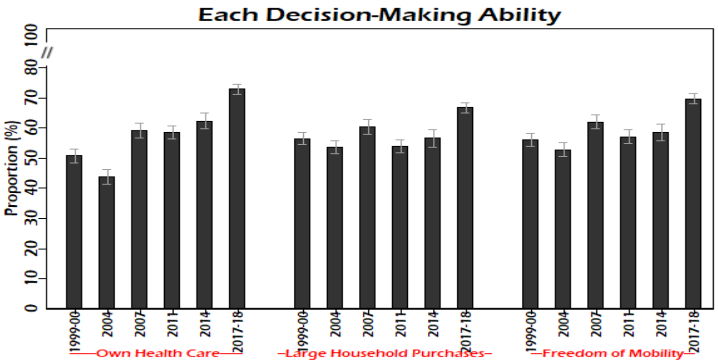

Abbreviation: BDHS: Bangladesh Demographic & Health Survey

The prevalence of autonomy about own health care increased from 50.8% (95% CI: 48.5-53.0) in 1999-00 to 72.8% (95% CI: 71.1-74.5) in 2017-18 (Figure 2). For major household purchases, it increased from 56.5% (95% CI: 54.4-58.5) in 1999-00 to 66.7% (95% CI: 65.0-68.4) in 2017-18. A similar change was observed for freedom of mobility.

Figure 3: Prevalence (95% CI) of 'lone' decision-making by survey, BDHS 1999-00 to 2017-18

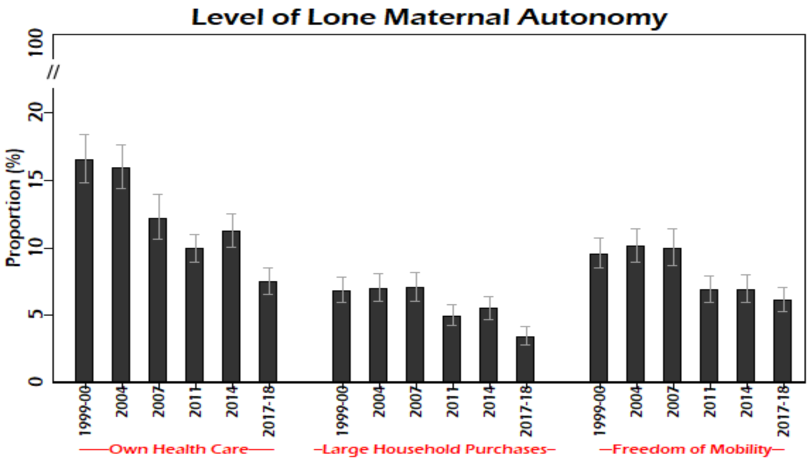

Abbreviation: BDHS: Bangladesh Demographic & Health Survey

The prevalence of lone autonomy declined in 2017-18 compared to its 1999-00 level (Figure 3). For instance, the proportion of mothers who could decide alone about their own health was 16.5% (95% CI: 14.8-18.4) in 1999-00 and 7.5% (95% CI: 6.6-8.5) in 2017-18. Similarly, the prevalence of lone autonomy for large household purchases and freedom of mobility declined.

Table 2: Prevalence (95% confidence interval) and trends of any maternal autonomy by sociodemographic characteristics

Variable	1999-00	2004	2007	2011	2014	2017-18	Mean change
**Maternal age (in year)**							
15-19	64.4 (60.8,67.8)	60.5 (56.7,64.2)	73.7 (69.7,77.3)	63.8 (60.2,67.3)	67.4 (63.5,71.1)	70.5 (67.0,73.7)	**1.2* (0.3, 2.1)**
20-29	73.6 (71.6,75.5)	68.7 (66.5,70.9)	79.0 (76.9,81.0)	72.2 (70.3,74.0)	72.2 (69.8,74.5)	84.9 (83.4,86.2)	**1.9*** (1.4,2.4)**
30-49	75.8 (72.6,78.8)	74.8 (71.3,78.0)	83.0 (79.6,86.0)	79.3 (76.0,82.2)	81.1 (77.7,84.1)	92.1 (90.1,93.8)	**2.9*** (2.2, 3.6)**
**Maternal education level**							
No education	70.2 (67.8,72.5)	65.8 (62.9,68.6)	74.3 (70.7,77.7)	67.9 (64.1,71.6)	71.4 (66.6,75.8)	83.1 (78.1,87.1)	**1.3* (0.3, 2.3)**
Primary	69.5 (66.5,72.3)	69.1 (66.0,72.0)	79.1 (76.2,81.7)	70.5 (67.7,73.1)	71.1 (67.6,74.4)	84.5 (82.3,86.5)	**2.2*** (1.5, 2.9)**
Secondary	76.2 (73.0,79.2)	67.5 (64.3,70.5)	79.4 (76.8,81.9)	71.9 (69.7,74.0)	73.3 (70.8,75.8)	83.0 (81.3,84.6)	**1.7*** (1.0, 2.3)**
College/above	87.7 (81.7,91.9)	81.8 (75.6,86.7)	87.1 (81.5,91.1)	85.0 (80.4,88.6)	78.5 (74.1,82.4)	85.3 (82.5,87.7)	0.3 (-0.3, 0.7)
**Paternal education level**							
No education	67.8 (65.3,70.2)	65.9 (63.1,68.6)	77.2 (74.2,79.9)	71.1 (68.1,73.9)	72.1 (68.0,75.8)	88.4 (85.5,90.8)	**2.8*** (2.0,3.5)**
Primary	72.8 (69.6,75.7)	68.3 (65.0,71.4)	78.0 (74.8,80.9)	70.3 (67.5,73.0)	72.1 (68.7,75.2)	85.6 (83.7,87.4)	**2.3*** (1.6, 2.9)**
Secondary	74.4 (71.1,77.4)	68.8 (65.4,72.1)	78.3 (75.1,81.2)	71.1 (68.3,73.7)	72.4 (69.5,75.2)	82.2 (80.1,84.1)	**1.5*** (0.8, 2.1)**
College/above	84.2 (80.1,87.7)	75.3 (70.3,79.7)	85.9 (81.3,89.5)	78.2 (74.3,81.7)	77.4 (73.4,81.0)	85.8 (83.1,88.2)	0.5 (-0.3, 1.4)
**Current work**							
No	70.7 (69.0,72.3)	66.6 (64.7,68.4)	77.3 (75.4,79.1)	70.9 (69.3,72.4)	71.8 (69.9,73.7)	82.7 (81.1,84.1)	**1.9*** (1.5, 2.4)**
Yes	79.1 (75.5,82.3)	76.5 (72.4,80.1)	83.2 (79.9,86.1)	82.1 (77.5,86.0)	76.6 (72.6,80.2)	85.7 (83.9,87.4)	**1.2*** (0.5, 1.8)**
**Wealth quintile**							
Poorest	67.4 (64.1,70.6)	67.2 (63.7,70.5)	76.2 (72.3,79.6)	73.0 (69.8,76.0)	66.7 (62.2,70.8)	86.3 (84.0,88.3)	**2.6*** (1.8, 3.5)**
Poorer	68.3 (64.8,71.7)	65.5 (61.6,69.3)	77.5 (73.8,80.7)	70.3 (66.7,73.6)	72.9 (69.2,76.4)	83.0 (80.4,85.4)	**2.6*** (1.8, 3.4)**
Middle	73.3 (69.8,76.5)	67.0 (63.1,70.7)	76.2 (72.1,79.9)	68.3 (64.8,71.5)	74.2 (69.8,78.2)	82.8 (80.0,85.4)	**1.9*** (1.0, 2.7)**
Richer	75.2 (71.4,78.5)	68.6 (64.6,72.4)	79.6 (75.7,82.9)	71.7 (68.4,74.9)	74.0 (70.3,77.4)	83.4 (80.7,85.8)	**1.6*** (0.7, 2.4)**
Richest	79.9 (76.6,82.8)	73.8 (70.2,77.1)	84.4 (81.0,87.2)	75.6 (72.2,78.7)	77.6 (74.2,80.7)	83.4 (80.6,85.9)	0.7 (-0.1, 1.5)
**Place of residence**							
Urban	75.9 (73.0,78.7)	75.5 (72.5,78.2)	83.4 (80.8,85.6)	78.0 (75.3,80.5)	77.4 (74.6,80.0)	85.8 (83.7,87.6)	**1.6*** (0.8, 2.3)**
Rural	71.2 (69.5,73.0)	66.4 (64.4,68.3)	77.3 (75.4,79.2)	69.9 (68.1,71.6)	71.4 (69.2,73.5)	83.1 (81.7,84.4)	**2.0*** (1.5, 2.5)**
**Overall**	72.0 (70.5,73.5)	68.2 (66.5,69.8)	78.6 (77.0,80.2)	71.8 (70.3,73.2)	73.0 (71.2,74.6)	83.8 (82.7,84.9)	**2.0*** (1.6, 2.4)**

*: $p<0.05$, **: $p<0.01$, ***: $p<0.001$

Table 2 shows the prevalence and trends of any maternal autonomy (i.e., at least one decision-making ability) according to selected background characteristics. Regardless of characteristics, the prevalence of autonomy increased significantly across most survey years. Overall, prevalence of any autonomy increased from 72.0% (95% CI: 70.5-73.5) in 1999-00 to 83.8% (95% CI: 82.7-84.9) in 2017-18. We also estimated the prevalence of high decision-making abilities (S2 Table); the trends were similar to Table 2.

Comparison of study sample by presence of any maternal autonomy

Factors Affecting Maternal Autonomy

Table 3: Factors associated with any maternal autonomy, BDHS 1999-00 to 2017-18

Variable		Unadjusted		Adjusted[1]	
		POR (95% CI)	p-value	POR (95% CI)	p-value
Maternal age (Ref: 15-19 years)	20-29	1.6*** (1.4,1.7)	<0.001	1.3*** (1.2,1.4)	<0.001
	30-49	2.3*** (2.0,2.5)	<0.001	2.1*** (1.8,2.4)	<0.001
Number of children (Ref: 1)	2-3	1.6*** (1.5,1.7)	<0.001	1.4*** (1.3,1.6)	<0.001
	4 or More	1.3*** (1.2,1.5)	<0.001	1.2** (1.1,1.4)	0.004
Maternal education (Ref: No formal education)	Primary	1.2*** (1.1,1.3)	<0.001	1.2*** (1.1,1.4)	<0.001
	Secondary	1.3*** (1.2,1.4)	<0.001	1.4*** (1.2,1.6)	<0.001
	College/above	2.2*** (1.9,2.5)	<0.001	1.9*** (1.6,2.2)	<0.001
Paternal education (Ref: No formal education)					
	Secondary	1.2** (1.1,1.3)	0.002	1.0 (0.9,1.1)	0.62
	College/above	1.7*** (1.5,1.9)	<0.001	1.1 (0.9,1.3)	0.28
Current work (Ref: No)	Yes	1.6*** (1.5,1.8)	<0.001	1.4*** (1.2,1.5)	<0.001
Religion (Ref: Muslim)	Other	1.0 (0.9,1.1)	0.85		
Mass media exposure (Ref: Not exposed)	Exposed	1.2*** (1.2,1.3)	<.001	1.1** (1.0,1.2)	0.004
Wealth quintile (Ref: Poorest)	Poorer	1.0 (0.9,1.1)	0.81	1.0 (0.9,1.1)	0.44
	Middle	1.0 (0.9,1.2)	0.49	0.9 (0.8,1.1)	0.36
	Richer	1.2* (1.0,1.3)	0.013	1.0 (0.8,1.1)	0.48
	Richest	1.4*** (1.2,1.6)	<.001	1.0 (0.8,1.1)	0.57
Place of residence (Ref: Urban)	Rural	0.7*** (0.6,0.8)	<.001	0.8*** (0.7,0.8)	<0.001
Division of residence (Ref: Dhaka)	Chittagong	0.9 (0.8,1.1)	0.31	0.9 (0.8,1.0)	0.18
	Rajshahi	1.0 (0.9,1.2)	0.88	1.1 (0.9,1.3)	0.24
	Khulna	0.9 (0.8,1.1)	0.39	1.0 (0.8,1.1)	0.6
	Barisal	0.8* (0.7,1.0)	0.032	0.8* (0.7,1.0)	0.028
	Sylhet	0.6*** (0.5,0.7)	<0.001	0.7*** (0.6,0.7)	<0.001
	Rangpur	1.2* (1.0,1.5)	0.029	1.2 (1.0,1.5)	0.063
	Mymensingh	2.0*** (1.4,2.7)	<0.001	1.2 (0.8,1.6)	0.40
Survey year (Ref: 1999-00)	2004	0.8** (0.7,0.9)	0.005	0.8*** (0.7,0.9)	<0.001
	2007	1.4*** (1.2,1.6)	<0.001	1.3*** (1.1,1.5)	<0.001

	2011	1.0 (0.9,1.1)	0.76	0.9 (0.8,1.0)	0.079
	2014	1.1 (0.9,1.2)	0.43	0.9 (0.8,1.0)	0.12
	2017-18	2.0*** (1.8,2.3)	<0.001	1.7*** (1.4,1.9)	<0.001

1.Adjusted for all variables in the column
Abbreviations: BDHS: Bangladesh Demographic & Health Survey; CI: Confidence interval; POR: Prevalence odds ratio

In Table 3, the factors associated with having any autonomy were reported. In the adjusted analysis, overall, mothers with older age, higher parity, and more education had higher odds of autonomy. For instance, compared to mothers with no formal education, those with primary (adjusted POR: 1.2, 95% CI: 1.1-1.4, p<0.001), secondary (adjusted POR: 1.4, 95% CI: 1.2-1.6, p<0.001), or college/above (adjusted POR: 1.9, 95% CI: 1.5-2.2, p<0.001) education had higher odds of having any autonomy. On the other hand, paternal education and household wealth status were not associated with maternal autonomy. We also performed several stratified analyses based on the number of decision-making abilities to compare distributions and associated factors, the associated factors were similar to those observed for any autonomy. We also compared the sample by presence maternal autonomy (S3 to S4 Tables). The factors were reported by the level of autonomy (i.e., low and high autonomy, S5 Table), these were similar to the factors reported in Table 3. .

Discussion

The prevalence of maternal autonomy increased significantly in Bangladesh over the survey years. This change was observed regardless of most sociodemographic and socioeconomic characteristics. This result supports our hypothesis regarding trends in the prevalence of autonomy. Older age, higher education, employment, and residence in urban regions or in certain administrative divisions were associated with higher autonomy.

The upward trend in the prevalence of maternal autonomy is a welcome finding of our study. Previous studies from other countries reported factors associated with maternal autonomy regarding health care [14–17]. Overall, those findings are consistent with our results, with mothers who are older or who

have higher education having a higher likelihood of decision-making ability about healthcare compared to younger or low-educated mothers.

Our findings regarding the association between maternal autonomy and age are consistent with past studies. As women get older, they develop confidence in their decision-making and problem-solving abilities, which allows them to negotiate more with their husbands about healthcare, household purchases, and mobility [15,16]. Younger mothers are more likely to be newly married and may live with their in-laws in the same household, where the in-laws may have more control over household decisions [14,27].

Similar to other studies, maternal education level had a positive relationship with autonomy [14–17]. Education increases confidence and opportunities to overcome external barriers by decreasing the power differential between mothers and other stakeholders, including husbands, family members, and health care providers. Educated mothers may have a greater understanding of their rights, and the importance of expressing their opinions than uneducated mothers. For instance, educated women may be more aware of the value of utilizing healthcare, which can allow them to talk with husbands about healthcare [28]. They can also obtain more information from public health campaigns and other knowledge sources (e.g., social media), therefore, can identify the appropriate time to seek health care [28–30]. Educated women generally have more earning opportunities, which can make them financially independent. They may also work outside the home, which ultimately can increase their freedom of mobility.

Mothers in urban regions and certain administrative divisions had significantly higher autonomy compared to those in rural regions [16]. In addition to individual (e.g., age) determinants, community-level determinants such as sociocultural/socioeconomic differences between regions in Bangladesh may play a role [31,32]. Urban residence may provide better opportunities for work, healthcare, and travel, which can decrease the reliance of women on others [7]. For example, the shorter distance between homes and hospitals in urban regions compared to rural regions allows women to seek healthcare on their own. Urban regions often have better transportation facilities than rural regions, which can reduce transportation barriers.

Additionally, urban residents are often more educated than rural residents, which can contribute to the creation of a sociocultural environment that allows women more decision-making power [7,33]. Earlier reports have shown socioeconomic (e.g., education level) and sociocultural differences by geographic divisions, which can also contribute to divisional differences in autonomy [7,8,34]. These differences may also contribute to disparities in healthcare utilization and outcomes between rural-urban places and divisions in Bangladesh [7].

Contrary to our hypothesis, paternal education and household wealth were not associated with maternal autonomy. Household wealth was computed by principal component analysis of basic household construction materials and household belongings, and did not necessarily indicate maternal earning power or income, which may explain its lack of association [7]. Women in richer families may not need to work to earn money, which can also negatively impact maternal autonomy [35]. Earlier studies showed similar results regarding association with women's own education while lack of association with husband's education or household wealth [15,16,36,37]. On the other hand, the positive association of maternal autonomy with education and employment indicates the importance of 'individual' socioeconomic variables and of creating more educational and earning opportunities for mothers [16,18].

There may not be direct measures or indicators for maternal autonomy [4], however, we used a simple definition with three variables present in every BDHS [7–11,38]. These variables provided insights about maternal autonomy in Bangladesh [7–11,38], and helped us to identify the mothers (e.g., low education or no work) who may have lower autonomy.

The positive association of maternal education or employment with their autonomy indicates that expanding educational and earning opportunities for women may increase their autonomy. More public campaigns should be carried out to increase awareness about maternal autonomy, education, and healthcare utilization/outcomes in Bangladesh. Moreover, supporting community-based programs to increase education levels would not only increase maternal autonomy but can also contribute to overall health, social, and

economic developments [14–17]. This could also reduce other disparities in health care utilization and outcomes. The dearth of studies on trends/factors affecting maternal autonomy also signifies the importance of future quantitative (e.g., to understand the impact of other socioeconomic or sociocultural factors) and qualitative research (e.g., to understand the perspectives of husbands, in-laws, and other family members on the role of autonomy).

This study has several notable strengths. First, this was a large, nationally representative study covering rural and urban regions of all administrative divisions in Bangladesh. Covering all regions made these findings generalizable to the country. BDHS used a standardized and validated questionnaire that also increased the authenticity of our findings. The response rate of the survey was high. This analysis was also conducted after accounting for all available confounders.

The limitations of the study also warrant discussion. First, we investigated the associations in multiple individual cross-sectional surveys, meaning that BDHS collected information on all variables at the same time during each study wave. Therefore, any observed association between exposures and outcomes may not be causal. The data were collected based on self-reports, that may vary from actual decision-making, and there may be some overreporting to please the interviewers (i.e., social desirability bias) [5]. The data included up to three years since the birth, autonomy may differ from the period immediately after birth.

Conclusions

Although maternal autonomy's prevalence has increased substantially during the past two decades in Bangladesh, a significant proportion of mothers still do not have any autonomy. In a patriarchal society like Bangladesh where husbands are the primary decision-makers of families, it may require a long time to improve the autonomy of mothers and change sociocultural norms, including attitudes towards maternal autonomy. Considering maternal autonomy' positive association with education and work, creating more educational or earning opportunities can increase maternal autonomy. More qualitative and quantitative studies are also required to understand the way to improve it.

Declarations

Acknowledgment

This manuscript was a part of the doctoral dissertation project of the primary author (i.e., Gulam Kibria). The primary author thanks his mentors, teachers, friends, family members, and other well-wishers for their support; he also thanks the ICF International for the approval to use the data as well as the survey participants for their time. While no funding was received for this study, the author acknowledges the fundings received from the University of Maryland, Baltimore and Johns Hopkins University for his doctoral education.

Consent for publication: Not applicable

Availability of data and material: Data is available from https://dhsprogram.com/data/available-datasets.cfm

Authors contributions

Conceptualization: Gulam Muhammed Al Kibria, Jon Mark Hirshon, Jennifer Albrecht

Data Cleaning and Analysis: Gulam Muhammed Al Kibria

Investigation: Gulam Muhammed Al Kibria, Jon Mark Hirshon, Jennifer Albrecht, Wendy Lane, Kristen Stafford, Laundette Jones, and Roumen Vesselinov

Methodology: Gulam Muhammed Al Kibria, Jon Mark Hirshon, Jennifer Albrecht, Kristen Stafford, and Roumen Vesselinov

Writing- Original Draft: Gulam Muhammed Al Kibria

Writing – Review & Editing: Jon Mark Hirshon, Jennifer Albrecht, Wendy Lane, Kristen Stafford, Laundette Jones, and Roumen Vesselinov

Supervision: Jon Mark Hirshon, Jennifer Albrecht, Wendy Lane, Kristen Stafford, Laundette Jones, and Roumen Vesselinov

Competing interests: The authors have declared that no competing interests exist.

References

1. Ghose B, Feng D, Tang S, Yaya S, He Z, Udenigwe O, et al. Women's decision-making autonomy and utilisation of maternal healthcare services: results from the Bangladesh Demographic and Health Survey. BMJ Open. 2017;7: e017142. doi:10.1136/bmjopen-2017-017142

2. Kishor S. A Focus on Gender: Collected Papers on Gender Using DHS Data. Calverton, Maryland, USA: ORC Macro; 2005. Available: https://dhsprogram.com/pubs/pdf/OD32/OD32.pdf

3. Ndaimani A, Mhlanga M, Dube-Mawerewere V. The Association between Women's Empowerment and Uptake of Child Health Services: A Demographic and Health Survey-Based Synthesis. Rockville, Maryland, USA: ICF; 2021. Available: https://dhsprogram.com/pubs/pdf/WP139/WP139.pdf

4. Haider MR, Qureshi ZP, Khan MM. Effects of women's autonomy on maternal healthcare utilization in Bangladesh: Evidence from a national survey. Sex Reprod Healthc. 2017;14: 40–47. doi:10.1016/j.srhc.2017.09.002

5. Haque SE, Rahman M, Mostofa MdG, Zahan MdS. Reproductive Health Care Utilization among Young Mothers in Bangladesh: Does Autonomy Matter? Womens Health Issues. 2012;22: e171–e180. doi:10.1016/j.whi.2011.08.004

6. Sharma A, Kader M. Effect of Women's Decision-Making Autonomy on Infant's Birth Weight in Rural Bangladesh. ISRN Pediatr. 2013;2013: 1–8. doi:10.1155/2013/159542

7. National Institute of Population Research and Training (NIPORT), Mitra and Associates, ICF International. Bangladesh Demographic and Health Survey 2017-18. Dhaka, Bangladesh; 2020.

8. National Institute of Population Research and Training (NIPORT), Mitra and Associates, ICF International. Bangladesh Demographic and Health Survey 2014. Dhaka, Bangladesh; 2015.

9. National Institute of Population Research and Training (NIPORT), Mitra and Associates, ICF International. Bangladesh Demographic and Health Survey 2011. Dhaka, Bangladesh; 2013. Available: https://dhsprogram.com/pubs/pdf/fr265/fr265.pdf

10. National Institute of Population Research and Training (NIPORT), Mitra and Associates, ICF International. Bangladesh Demographic and Health Survey 1999-00. Dhaka, Bangladesh; 2013. Available: https://dhsprogram.com/pubs/pdf/fr265/fr265.pdf

11. National Institute of Population Research and Training (NIPORT), Mitra and Associates, ICF International. Bangladesh Demographic and Health Survey 2004. Dhaka, Bangladesh; 2013. Available: https://dhsprogram.com/pubs/pdf/fr265/fr265.pdf

12. National Institute of Population Research and Training (NIPORT), Mitra and Associates, ICF International. Bangladesh Demographic and Health Survey 1993-94. Dhaka, Bangladesh; 2013. Available: https://dhsprogram.com/pubs/pdf/fr265/fr265.pdf

13. The World Bank. Bangladesh GDP Per Capita 1962-2022. [cited 1 Jun 2022]. Available: https://www.macrotrends.net/countries/BGD/bangladesh/gdp-per-capita

14. Senarath U, Nalika Sepali Gunawardena. Women's Autonomy in Decision Making for Health Care in South Asia. Asia Pac J Public Health. 2009;21: 137–143. doi:10.1177/1010539509331590

15. Asabu MD, Altaseb DK. The trends of women's autonomy in health care decision making and associated factors in Ethiopia: evidence from 2005, 2011 and 2016 DHS data. BMC Womens Health. 2021;21: 371. doi:10.1186/s12905-021-01517-9

16. Acharya DR, Bell JS, Simkhada P, van Teijlingen ER, Regmi PR. Women's autonomy in household decision-making: a demographic study in Nepal. Reprod Health. 2010;7: 15. doi:10.1186/1742-4755-7-15

17. Ebrahim NB, Atteraya MS. Women's Decision-Making Autonomy and their Attitude towards Wife-Beating: Findings from the 2011 Ethiopia's Demographic and Health Survey. J Immigr Minor Health. 2018;20: 603–611. doi:10.1007/s10903-017-0592-6

18. Abedin S, Arunachalam D. Risk Deciphering Pathways from Women's Autonomy to Perinatal Deaths in Bangladesh. Matern Child Health J. 2022;26: 2339–2345. doi:10.1007/s10995-022-03477-4

19. Adhikari R. Effect of Women's autonomy on maternal health service utilization in Nepal: a cross sectional study. BMC Womens Health. 2016;16: 26. doi:10.1186/s12905-016-0305-7

20. Khan JR, Awan N. A comprehensive analysis on child mortality and its determinants in Bangladesh using frailty models. Arch Public Health. 2017;75: 58. doi:10.1186/s13690-017-0224-6

21. Khan RJ, Stewart CP, Christian P, Schulze KJ, Wu L, LeClerq SC, et al. A cross-sectional study of the prevalence and risk factors for hypertension in rural Nepali women. BMC Public Health. 2013;13. doi:10.1186/1471-2458-13-55

22. Nisar YB, Dibley MJ. Determinants of neonatal mortality in Pakistan: secondary analysis of Pakistan Demographic and Health Survey 2006–07. BMC Public Health. 2014;14: 663. doi:10.1186/1471-2458-14-663

23. Titaley CR, Dibley MJ, Agho K, Roberts CL, Hall J. Determinants of neonatal mortality in Indonesia. BMC Public Health. 2008;8: 232. doi:10.1186/1471-2458-8-232

24. Rahman MM, Ara T, Mahmud S, Samad N. Revisit the correlates of infant mortality in Bangladesh: findings from two nationwide cross-sectional studies. BMJ Open. 2021;11: e045506. doi:10.1136/bmjopen-2020-045506

25. Ghose B, Feng D, Tang S, Yaya S, He Z, Udenigwe O, et al. Women's decision-making autonomy and utilisation of maternal healthcare services: results from the Bangladesh Demographic and Health Survey. BMJ Open. 2017;7: e017142. doi:10.1136/bmjopen-2017-017142

26. Stata Corporation, College Station, Texas USA. StataCorp. 2017. 2017 [cited 8 May 2017]. Available: https://www.stata.com/support/faqs/resources/citing-software-documentation-faqs/

27. Rajaram R, Perkins JM, Joe W, Subramanian SV. Individual and community levels of maternal autonomy and child undernutrition in India. Int J Public Health. 2017;62: 327–335. doi:10.1007/s00038-016-0850-8

28. Shahjahan M, Chowdhury HA, Al-Hadhrami AY, Harun GD. Antenatal and postnatal care practices among mothers in rural Bangladesh: A community based cross-sectional study. Midwifery. 2017;52: 42–48. doi:10.1016/j.midw.2017.05.011

29. Roy S, Haque MdA. Effect of antenatal care and social well-being on early neonatal mortality in Bangladesh. BMC Pregnancy Childbirth. 2018;18: 485. doi:10.1186/s12884-018-2129-y

30. Saad–Haddad G, DeJong J, Terreri N, Restrepo–Méndez MC, Perin J, Vaz L, et al. Patterns and determinants of antenatal care utilization: analysis of national survey data in seven countdown countries. J Glob Health. 2016;6: 010404. doi:10.7189/jogh.06.010404

31. Auer S. Factors Associated with Reproductive Autonomy: A Social Ecological Approach. Emory University. 2020. Available: https://etd.library.emory.edu/concern/etds/0r967473t?locale=es

32. Centers for Disease Control and Prevention. The Social-Ecological Model. Jan 2022. Available: https://www.cdc.gov/violenceprevention/about/social-ecologicalmodel.html

33. Bangladesh Bureau of Statistics. 2011 Population and Housing Census of the People's Republic of Bangladesh. 2014. Available: http://203.112.218.65:8008/WebTestApplication/userfiles/Image/National%20Reports/Population%20%20Housing%20Census%202011.pdf

34. Kalam A. Social Norms and Impediments of Women Development in Bangladesh. Int J Soc Sci Stud. 2014;2: 100–109. doi:10.11114/ijsss.v2i2.365

35. Islam MdS, Islam S, Fatema K, Khanum R. Rural women participation in farm and off-farm activities and household income in Bangladesh. Heliyon. 2022;8: e10618. doi:10.1016/j.heliyon.2022.e10618

36. Bou Malhab S, Sacre H, Malaeb D, Lahoud N, Khachman D, Azzi J, et al. Factors related to autonomy among Lebanese women: a web-based cross-sectional study. BMC Womens Health. 2021;21: 369. doi:10.1186/s12905-021-01501-3

37. Adhikari R, Sawangdee Y. Influence of women's autonomy on infant mortality in Nepal. Reprod Health. 2011;8: 7. doi:10.1186/1742-4755-8-7

38. National Institute of Population Research and Training (NIPORT), Mitra and Associates, ICF International. Bangladesh Demographic and Health Survey 2007. Dhaka, Bangladesh; 2013. Available: https://dhsprogram.com/pubs/pdf/fr265/fr265.pdf

Click here to access/download
Supporting Information
Supplemental_Material_06232023_jsa.docx

Ethical approval document

Click here to access/download

Supporting Information

HRP-518 - Not Human Research Determination.pdf
