# Supplementary material for: Prevalence, trends, and factors associated with maternal autonomy regarding healthcare, finances, and mobility in Bangladesh: Analysis of Demographic and Health Surveys 1999–2018
Source: PLOS Glob Public Health. 2024 Feb 2;4(2):e0002816. doi: 10.1371/journal.pgph.0002816 (PMC10836669; doi:10.1371/journal.pgph.0002816)
Supplement: Attachment — Submitted filename: Rebutal_letter_GMAK_21Aug2023.docx [file pgph.0002816.s007.docx]

Gulam Muhammed Al Kibria, MBBS, MSPH, PhD

Johns Hopkins University Bloomberg School of Public Health

615 N Wolfe Street, Baltimore, MD 21205

443-477-9403, gkibria1@jhu.edu

**Associate Editor**

**PLoS Global Public Health**

Nov 11, 2023

Dear Editor:

This is to inform you that we have prepared the revised version of the manuscript on ‘**Prevalence, Trends, and Factors Associated with Maternal Autonomy Regarding Healthcare, Finances, and Mobility in Bangladesh: Analysis of Demographic and Health Surveys 1999-2018’** for your consideration to publish in the *PLoS* *Global Public Health Journal*. Here we have provided the itemized responses

**Reviewer #1:**

**Comment 1:** Aside from various edits related to further explanation of points raised by the authors, the need for more narrative explanation of results and background research, as well as additional references to the specific context [in the conclusion], the draft requires slight adjustment to the conclusions presented. Following on from the stated hypothesis, ("the prevalence of maternal autonomy has increased and that women with higher socioeconomic status (e.g., education or wealth) have higher autonomy than their counterparts") the data displayed in Table 3, particularly relating to "wealth quintile" demonstrate that none of the adjusted figures are significantly associated with maternal autonomy. However, in your conclusion section (page 12), you state that your findings confirm your hypothesis. While I can take this to understand that the first part of the hypothesis related to a prevalence increase has been supported, the second part of the hypothesis has clearly been demonstrated to have mixed results. It is critical that this is adequately addressed.

***Response:*** *Thank you for the comment, we agree with you and have edited the part. We have rewritten the first paragraph of the discussion as follows:* “The prevalence of maternal autonomy increased significantly in Bangladesh over the survey years and this change was observed regardless of most sociodemographic and socioeconomic characteristics; this result supports our hypothesis regarding trends in the prevalence of autonomy. Older age, higher education, employment, and residence in urban regions or certain administrative divisions were associated with higher autonomy; this result also supports part of our hypothesis about the association of socioeconomic status with the outcome.

*”*

**Comment 2:** Additionally, the inclusion of the data on "lone maternal autonomy" is not thoroughly addressed anywhere other than the brief section in the results. Please consider adding more context to these findings as they represent an important facet of your argument.

***Response:*** *Thank you for noticing this, we agree with you and have added the following paragraph: “*When we examined the trend of ‘lone’ decision-making ability, we observed a downward trend. Ghose suggested that ‘autonomy’ without husbands’ involvement can result in poor spousal communications and low involvement of male partners in maternal health care utilization [25]. Previous studies from Bangladesh reported that a lower involvement of males is also linked to poor reproductive outcomes and low maternal health care utilization [39,40]. Taking the importance of ‘joint’ autonomy into account, the ‘downward trend’ of lone decision-making ability or the upward trend of ‘joint’ decision-making ability may be an indication of the improvement of spousal relationships, including engagement of men in women’s decisions.

*”*

**Reviewer #2:**

**Comment 1:** This manuscript reports on the trends in prevalence and factors associated with maternal autonomy using a common definition from DHS in Bangladesh. This manuscript is well-written and provides insight into how maternal autonomy might be defined; these findings could be of great interest to health economists and gender/health experts. A few comments to consider:

***Response:*** *Thank you for the comment. We appreciate your reviews.*

**Comment 2:** Abstract: It would be helpful to add some numbers to show the increase in maternal autonomy, rather than just " While the level of maternal autonomy has increased over the past two decades,..."

***Response:*** *Thank you for noticing this, we agree with you and have added that as below “While the level of maternal autonomy has increased over the past two decades (i.e., from 72.0% in 1999-00 to 83.8% in 2017-18), a substantial proportion of mothers still do not have autonomy.”*

**Comment 3:** Introduction: Please add the number of surveys providing data (6?) when describing the overall study objective.

***Response:*** *Thank you for the comment, we have edited the part.*

**Comment 4:** Methods: It would be helpful to state why only included women who had given birth within three years preceding the survey.

***Response:*** *Thank you for the comment, we have edited the part.*

**Comment 5:** Other variables (page 5); please provide rationale for the other variables that were selected.

***Response:*** *Thank you for the comment, we have edited the part.*

**Comment 6:** It would be useful to reference "freedom of mobility" when discussing the third variable described.

***Response:*** *Thank you for the comment, we have edited the part.*

**Comment 7:** Results: What is the relationship between division of residence and place of residence? It seems that Dhaka and Chittagong would be the "urban" sample and the other districts would be "rural".

***Response:*** *Thank you for the comment, rural and urban regions were drawn from each division separately, including Dhaka and Chittagong divisions. Different divisions have different educational and employment opportunities to impact the autonomy.*

**Comment 8:** Discussion: I would love to see a larger discussion of regional differences in results and rationale for why that might be happening. Perhaps another area of future exploration would be to look at this issue within districts that were not included in DHS?

***Response:*** *Thank you for the comment, we have edited the part. Added the following sentences: “*There may be intra-divisional differences due to socioeconomic/sociocultural differences between districts of each division [7]. Future studies should explore the divisional differences and may include districts in the analysis.*”*

**Comment 9:** I question the idea that public campaigns will somehow be effective in addressing this long-standing issue of women's empowerment within a patriarchal social structure. The authors may consider removing the line from the discussion and/or justify why it might be a tenable way to address the issue at hand.
"More public campaigns should be carried out to increase awareness about maternal autonomy, education, and healthcare utilization/outcomes in Bangladesh".

***Response:*** *Thank you for the comment, we have removed the part as you suggested.*

**Comment 10:** Conclusion: The authors state that " it may require a long time to improve the autonomy of mothers and change sociocultural norms, ...". Given that they are reporting on an approximate 20 year period, I wonder if they might want to reframe this point in the positive. Twenty years for significant changes in women's empowerment seems like good progress to me.

***Response:*** *We have edited the sentence as you suggested, thank you again! Here is the edited part “*In a patriarchal society like Bangladesh where husbands are the primary decision-makers of families, it may require some time to improve the autonomy of mothers and change sociocultural norms, including attitudes towards maternal autonomy; however, our data suggest that it is possible to improve.*”*

Dear Editor, we believe we were able to address all the comments. We are looking forward to your decision.

Sincerely,

Gulam Muhammed Al Kibria, MBBS, MSPH, PhD
