# Supplementary material for: Prevalence, trends, and factors associated with maternal autonomy regarding healthcare, finances, and mobility in Bangladesh: Analysis of Demographic and Health Surveys 1999–2018
Source: PLOS Glob Public Health. 2024 Feb 2;4(2):e0002816. doi: 10.1371/journal.pgph.0002816 (PMC10836669; doi:10.1371/journal.pgph.0002816)
Supplement: Attachment — Submitted filename: Rebuttal letter Revision 2.docx [file pgph.0002816.s009.docx]

Gulam Muhammed Al Kibria, MBBS, MSPH, PhD

Johns Hopkins University Bloomberg School of Public Health

615 N Wolfe Street, Baltimore, MD 21205

443-477-9403, gkibria1@jhu.edu

**Associate Editor**

**PLoS Global Public Health**

Dec 12, 2023

Dear Editor:

This is to inform you that we have prepared the revised version of the manuscript on ‘**Prevalence, Trends, and Factors Associated with Maternal Autonomy Regarding Healthcare, Finances, and Mobility in Bangladesh: Analysis of Demographic and Health Surveys 1999-2018’** for your consideration to publish in the *PLoS* *Global Public Health Journal*. Here we have provided the itemized responses

**Comment 1:** Define earlier “Maternal autonomy is an indicator of overall socioeconomic development.”

Response: Thanks for the suggestion, we have made the changes.

**Comment 2**: Not sure it’s appropriate to group them together “low- and middle-income countries”

Response: Thank you for the comment, it is very common to group these countries.

**Comment 3:** Create a more narrative way to explain all of these findings, currently reading too much like a list - . Another study by Haque et al. (2012) had similar results.

Response: Edited the sentence, thanks for the comment.

**Comment 4:** Do they definitively show this or is this a more flexible conclusion the researchers drew? if the latter, speak to your teams interest in adding to the literature “Overall, these studies demonstrated that higher maternal autonomy could increase healthcare utilization and decrease maternal/neonatal complications.”

Response: Removed the part.

**Comment 5:** Have these developments impacted women? considering the topic of interest is maternal autonomy, worth discussing how SES is related to MA in more depth “In recent decades, significant socioeconomic developments have taken place in Bangladesh”

Response: Yes, edited the above sentence, thank you again!

**Comment 6:** this sentence first? “Studies from other countries suggest that socioeconomic status (e.g., education or income) could impact maternal autonomy [14–17].”

Response: thank you for the comment, it was placed here to make connections with the earlier part.

**Comment 7:** Why survey years in particular.

Response: We used these survey years because we wanted to have data on 2 decades to understand how it is changing in recent decades and most changes took place in past two decades, it is also important to mention that we did not have data from earlier years.

**Comment 8:** This paragraph seems to not make a lot of sense in context. suggest formulating a stronger thesis sentence

Response: agree with the comment, edited.

**Comment 9:** Why aren’t these categories evenly spaced, provide clearer explanation if possible or note that these were the preset categories from the survey “maternal age (i.e., 15-19, 20-29, and 30-49 years)”

Response: As we stated before, we did the categorization based on literature search. We also did it to see the autonomy among teenage mothers.

**Comment 10:** how is work defined “current work status”. defined? “mass media exposure”

Response: We actually reported the definition in supplemental table 1.

**Comment 11:** would suggest explaining the table more narratively and including the table as a supplement – “Table 1”

Response: Thank you for the comment, we have edited the paragraph. However, for one thing, we respectfully disagree with the reviewer about removing the table, it is important to describe the sociodemographic of the study participants.

**Comment 12:** suggest using a stacked bar chart to display “Figure 1”

Response: Responses were not mutually exclusive, we consider that not using a stacked chart is better.

**Comment 13:** hmm so saw increase in SES but decrease in lone maternal autonomy across the board.

Response: Yes, we have provided the explanation in the discussion section.

**Comment 14:** given that you’ve displayed “lone MA” and saw an overall decrease, would argue it’s important to clarify what kind of autonomy you’re speaking to. Additionally if “lone MA” is not going to be looked at further / used in analysis and recommendations, would advise it is removed “characteristics, the prevalence of au- tonomy”

Response: Agree, removed that part, thank you for the suggestion!

**Comment 15:** need to define again “****”

Response: Added that, thanks!

**Comment 16:** same comment as above “ The prevalence of maternal autonomy increased significantly in Bangladesh over the survey years.”does it? wasn’t the hypothesis that MA would follow increases in SES? “his result supports our hypothesis regarding trends in the prevalence of autonomy”

Response: The increase in prevalence we observed supports the hypothesis regarding the trend.

**Comment 17:** The upward trend in the prevalence of maternal autonomy is a welcome finding of our study. Previous studies from other countries reported factors associated with maternal autonomy regarding healthcare “the connection between these two sentences isn’t clear” this paragraph needs some work “Overall, those findings are consistent with our results, with mothers who are older or who have higher education having a higher likelihood of decision-making ability about healthcare compared to younger or low-educated mothers.”

Response: Agree, we actually joined two paragraphs to make them clearer.

**Comment 18:** any data on age at first pregnancy/birth? might contribute to power in the household/MA “Our findings regarding the association between maternal autonomy and age are consistent with past studies. As women get older, they develop confidence in their decision-making and problem-solving abili- ties, which allows them to negotiate more with their husbands about healthcare, household purchases, and mobility [15,16]. ”

Response: We used the reference to support that.

**Comment 19:** is this all information specific to Bangladesh, other LMICs or in general? make sure to call back to original context “Educated women generally have more earning opportunities, which can make them financially independent. They may also work outside the home, which ultimately can increase their freedom of mobility.”

Response: Thank you for the comment, this is true for all LMICs.

**Comment 20:** maybe worth clarifying/adding something earlier talking about the difference between household wealth and maternal earning “Women in richer families may not need to work to earn money, which can also negatively impact maternal autonomy [35].”

Response: We edited the sentence, thanks!

**Comment 21:** recommend speaking about this with more probability rather than concrete - “Moreover, supporting community-based programs to increase educa- tion levels would not only increase maternal autonomy but can also contribute to overall health, social, and economic developments [14–17].”

Response: We edited the sentence, thanks!

**Comment 22:** listed somewhere? - “The response rate of the survey was high. This analysis was also conducted after accounting for all available confounders.”

Response: It was given in the supplemental table 1.

Dear Editor, we believe we were able to address all the comments. We are looking forward to your decision.

Sincerely,

Gulam Muhammed Al Kibria, MBBS, MSPH, PhD
